# In Vitro Anticancer Activity and Integrated Multi-Target Computational Analysis of Ylang-Ylang Essential Oil Against Breast Cancer

**DOI:** 10.3390/ijms27198557

**Published:** 2026-09-24

**Authors:** Mohamed Chebaibi, Amine Elbouzidi, Mohamed Taibi, Mounir Haddou, Salah-Eddine Chebaibi, Aziz Tikent, Aimad Allali, Tarik Moubchir, Ibrahim Mssillou, Chakib Nejjari, Abdellah Baraich, Abdelfattah El Moussaoui, Sanae Achour

**Affiliations:** 1 Higher Institute of Nursing Professions and Health Techniques, Fez 30000, Morocco; mohamed.chebaibi@usmba.ac.ma (M.C.);; 2 Biomedical and Translational Research Laboratory, Faculty of Medicine and Pharmacy of Fez, Sidi Mohamed Ben Abdellah University, Fez 30000, Morocco; 3 Laboratory for Agricultural Production Improvement, Biotechnology, and Environment (LAPABE), Faculty of Sciences, Mohammed First University, Oujda 60000, Morocco; 4 Laboratory of Bioresources, Biotechnology, and Sustainable Development (L2BD), Faculty of Sciences, Mohammed Premier University, Boulevard Mohamed VI, B.P. 717, Oujda 60000, Morocco; 5 Plant Biotechnology Team, Faculty of Sciences, Abdelmalek Essaadi University, Tetouan 93000, Morocco; 6 Laboratory of Agricultural Production Improvement, Biotechnology and Environment (LAPABE), Faculty of Sciences, Mohammed Premier University, Oujda 60000, Morocco; 7 Euromed University of Fes, UEMF, Fez 30000, Morocco

**Keywords:** ylang-ylang essential oil, breast cancer, cytotoxicity, structural modeling, molecular docking, ADMET, ER-α, EGFR, PARP1

## Abstract

Ylang-ylang essential oil (YEO), obtained from *Cananga odorata*, contains chemically diverse constituents with potential anticancer activity that warrants further investigation. This study investigated the anti-breast-cancer potential of YEO through an integrated experimental and computational approach. The essential oil was chemically characterized by gas chromatography–mass spectrometry, and its in vitro cytotoxic activity was evaluated against ER-positive MCF-7 and triple-negative MDA-MB-231 and MDA-MB-436 breast-cancer cell lines using the MTT assay. Peripheral blood mononuclear cells were included as a preliminary model for assessing cytotoxic selectivity toward non-tumoral cells. To generate hypotheses concerning possible molecular interactions, identified constituents were evaluated against three breast-cancer-relevant targets: estrogen receptor alpha (ER-α), epidermal growth factor receptor (EGFR), and poly(ADP-ribose) polymerase 1 (PARP1). The computational workflow included a structural confidence assessment based on AlphaFold 2, together with CNN-assisted Gnina docking, Glide docking, and in silico ADMET profiling. The in vitro experiments showed that YEO had biological activity against breast-cancer cells, and the computational analyses showed that the ligand profiles were dependent on the target. Gnina identified trans-geranylgeraniol, farnesyl-decanoate and linalool as the top candidates for ER-α, EGFR and PARP1, respectively, while Glide pointed out δ-cadinene for ER-α, benzyl salicylate for EGFR and benzyl benzoate for PARP1. Both benzyl benzoate and α-cadinol also had potentially favorable multi-target binding profiles. The ADMET analysis found that several components had acceptable drug-likeness, but the high lipophilicity of some sesquiterpenes might limit their pharmacokinetic behavior. Overall, the results support the prioritization of selected YEO constituents as candidates for further investigation of potential multi-target anticancer activity. Nevertheless, the docking results remain predictive and do not demonstrate direct target inhibition or molecular synergy. Further biochemical, mechanistic, pharmacokinetic, and in vivo studies are needed to evaluate the biological relevance and translational potential of the prioritized constituents.

## 1. Introduction

The ylang-ylang tree (*Cananga odorata* Hook. F. & Thomson) is a tropical plant species which is well known in Southeast Asia. The essential oil obtained from its fragrant flowers is complex and has biological activity. In traditional folk medicine, ylang-ylang essential oil (YEO) has been used for its calming and antimicrobial properties. Currently, YEO is receiving increasing attention in the field of cancer research [1]. The oil’s chemical composition consists of monoterpenes, sesquiterpenes and phenylpropanoids, with important components including linalool, geraniol, methyl benzoate and germacrene-D [2,3]. Recent studies have underlined the strong anticancer potential of YEO, demonstrating that it is able to disrupt cancer pathways and cause cell death in a variety of cancer cell types [4,5].

Research currently shows that YEO has anticancer effects by means of a number of well-established molecular mechanisms. A key study carried out in 2024 found that C. odorata affects NOTCH1 signaling, an important pathway involved in the development of non-melanoma skin cancer, in A431 cells [6]. Moreover, YEO causes apoptosis and leads to cell cycle arrest, usually at the G2/M stage, by inducing DNA damage and by controlling the expression of pro-apoptotic genes such as TP53 [2,7]. In animal studies, for example in mice with Ehrlich Ascites Carcinoma (EAC), treatment with YEO greatly reduced tumor growth and increased survival rates, thus underscoring its potential as a systemic therapeutic agent [8].

It is the main phytochemicals in YEO that are responsible for its biological activity. The two major monoterpenes in the oil, linalool and geraniol, have been thoroughly studied for their ability to induce cell death via reactive oxygen species (ROS) and to inhibit angiogenesis [9,10]. Liriodenine, an alkaloid from *C. odorata*, has shown significant antitumor effects by inhibiting Bcl-2 and causing apoptosis in lung and colon cancer cells [11]. Moreover, sesquiterpenes such as β-caryophyllene increase the sensitivity of resistant cancer cells to conventional treatments [12].

Actually, nanotechnology is being used in pharmacological approaches in order to improve the delivery and effectiveness of YEO. Researchers have developed lipid-based nanovesicles and niosomes that are pH-sensitive and can be used for the co-delivery of YEO and chemotherapy drugs such as oxaliplatin [2,13]. By specifically directing these delivery systems to the tumor microenvironment, adverse effects are reduced and the overall efficiency of combination therapy is improved [2]. This kind of progress marks a significant step toward incorporating natural bioactive compounds into modern cancer treatments.

Although previous studies have reported anticancer effects of ylang-ylang essential oil and have explored nanoformulations for improving its delivery, important aspects of the relationship between its chemical composition, cell-type-dependent cytotoxicity, and potential molecular targets remain insufficiently integrated. The novelty of the present study is therefore not the first demonstration of anticancer activity or the development of a YEO nanoformulation. Instead, we combined GC–MS profiling of the tested oil with cytotoxicity assessment in ER-positive and triple-negative breast-cancer cell models, together with a preliminary selectivity assessment in PBMCs. We then evaluated all identified constituents against three breast-cancer-relevant targets—ER-α, EGFR, and PARP1—using complementary Gnina and Glide docking approaches, AlphaFold 2-based structural-confidence assessment, and in silico ADMET/drug-likeness profiling. This integrated workflow was used to prioritize candidate constituents and generate testable mechanistic hypotheses that can guide subsequent biochemical, mechanistic, and in vivo studies.

Based on these considerations, the current study examines the in vitro activity of ylang-ylang essential oil against breast-cancer cell lines and integrates chemical profiling with a target-oriented computational analysis of its constituents. GC–MS was used to characterize the tested oil, while cytotoxicity was evaluated in ER-positive MCF-7 and triple-negative MDA-MB-231 and MDA-MB-436 cells, with PBMCs included as a preliminary model of non-tumoral selectivity. The identified constituents were evaluated against ER-α, EGFR, and PARP1 using complementary CNN-assisted Gnina and Glide docking approaches, together with AlphaFold 2-based structural-confidence assessment and in silico ADMET/drug-likeness profiling. The aim was to prioritize candidate molecules and generate mechanistic hypotheses for further biochemical and in vivo investigation. These computational analyses are predictive and are not intended to establish direct target inhibition or clinical efficacy.

## 2. Result and Discussion

### 2.1. Chemicals Profiling by GC–MS

The GC–MS analysis identified 35 constituents in the YEO (Figure 1), representing a rich terpenoid mixture dominated by sesquiterpene hydrocarbons. The major compounds are summarized in (Table 1).

The chemical profile is dominated by sesquiterpene hydrocarbons (approximately 56% of total composition), with β-caryophyllene (15.17%), γ-cadinene (13.22%), germacrene D (11.97%), α-farnesene (6.45%), and α-humulene (4.83%) as the principal components. This sesquiterpene-rich profile is consistent with recent reports on ylang-ylang essential oil from various geographical origins [14,15]. A 2026 study by Bireller et al. confirmed that YEO consists of a terpenoid-rich mixture (99.31%) with germacrene D and β-caryophyllene as dominant constituents and demonstrated that malignant cells showed greater functional sensitivity than non-malignant cells [16]. The relatively high proportion of β-caryophyllene (15.17%) is particularly significant, as this compound is recognized as a selective agonist of the Cannabinoid Receptor Type 2 (CB2), the only known endogenous G-protein-coupled receptor selective for terpenoids [12].

### 2.2. Cytotoxicity Against Breast Cancer Cell Lines

The MTT assay was conducted over a 72-h treatment period with YEO concentra-tions ranging from 0.375 to 100 μg/mL. The cell viability and IC50 values obtained are presented in Figure 2 and Table 2. Two-way ANOVA showed significant effects of concentration, cell type and their interaction on cell viability for both YEO (F (7,64) = 1351.0, F (3,64) = 1457.8 and F (21,64) = 96.6, respectively; all *p* < 0.0001) and cisplatin (F (7,64) = 682.5, F (3,64) = 41.3 and F (21,64) = 12.2; all *p* < 0.0001). Compared with PBMCs exposed to the same concentration, YEO significantly reduced the viability of MCF-7 and MDA-MB-436 cells at every concentration from 1.5 µg/mL upwards (*p* < 0.0001) and that of MDA-MB-231 cells from 3 µg/mL upwards (*p* < 0.05 at 3 µg/mL, *p* < 0.001 at 10 µg/mL and *p* < 0.0001 at ≥30 µg/mL) (Figure 2A). In contrast, the viability of cisplatin-treated cancer cells differed markedly from that of PBMCs only at 25 and 50 µg/mL (*p* < 0.0001 for the three cell lines), with smaller and less consistent differences between 1 and 10 µg/mL (Figure 2B).

YEO was most potent against the hormone-receptor-positive MCF-7 cells (IC50 = 4.03 μg/mL), with an IC50 approximately two-fold lower than that of cisplatin (7.68 μg/mL; *p* = 0.005, extra sum-of-squares F-test). YEO was also active against the triple-negative MDA-MB-436 cell line (IC50 = 5.87 μg/mL), although its IC50 was moderately but significantly higher than that of cisplatin (3.86 μg/mL; *p* = 0.043); the IC50 values of YEO for MCF-7 and MDA-MB-436 cells did not differ significantly after Bonferroni correction (*p* = 0.22). Notably, the MDA-MB-231 cell line was significantly less sensitive to YEO (IC50 = 59.15 μg/mL) than MCF-7 and MDA-MB-436 cells (both *p* < 0.0001), and its IC50 was markedly higher than that of cisplatin (4.68 μg/mL; *p* < 0.0001), indicating cell-type-dependent differences in the metabolic viability response under the tested conditions. The basis of these differences was not determined by the present MTT assay.

The differential sensitivity among the three breast cancer cell lines is consistent with recent results reported in the literature. As shown in a systematic review by Mustapa et al. (2022), essential oils and the terpenoids they contain have very good anti-breast cancer pharmacological properties, their IC50 values usually being in the low μg/mL range [17]. The high level of activity displayed by YEO towards MCF-7 cells is in keeping with the well-known cytotoxic effect of β-caryophyllene, which has been found to cause apoptosis in MCF-7 cells by inducing G0/G1 cell cycle arrest and by altering the Bax/Bcl-2 ratio [12]. According to a thorough review by Ahmed in 2025, β-caryophyllene has IC50 values between 19 and 64 μM against different cancer cell lines, whereas its IC50 values remain above 100 μM for normal cells, thus showing its natural selectivity [12].

The fact that MDA-MB-231 cells show lower sensitivity (with an IC50 of 59.15 μg/mL) than MCF-7 and MDA-MB-436 is noteworthy. This could be due to the different molecular characteristics of triple-negative breast cancer (TNBC), a type of breast cancer that does not have estrogen, progesterone, or HER2 receptors and therefore depends on other survival pathways. Yet a key 2023 study by Sedky et al. showed that YEO, when delivered together with oxaliplatin in pH-sensitive lipid-based nanovesicles, greatly increased its cytotoxic effect on MDA-MB-231 cells [2]. This indicates that, although YEO on its own may be less effective against MDA-MB-231 cells, its components are able to make these cells more sensitive to conventional chemotherapy, possibly by affecting inflammatory signaling pathways such as COX-2 and STAT3 [12].

The fact that YEO exhibited high cytotoxicity against MDA-MB-436 cells (IC50 = 5.87 μg/mL) is noteworthy because this cell line carries a BRCA1 mutation and has impaired homologous recombination repair. However, the present viability data do not establish whether this response is dependent on p53 status or mediated by p53-independent mechanisms. Mitochondrial dysfunction, oxidative stress, DNA-damage accumulation, and activation of alternative apoptotic pathways may represent possible explanations, but these mechanisms were not directly investigated in the present study. Therefore, these interpretations should be considered hypotheses rather than demonstrated mechanisms. Further studies measuring p53 expression and activity, mitochondrial membrane potential, reactive oxygen species, caspase activation, DNA-damage markers, and the effects of pathway-specific inhibitors or genetic perturbations are required to define the mechanism underlying YEO-induced cytotoxicity in MDA-MB-436 cells.

The fact that YEO exhibited high cytotoxicity against MDA-MB-436 cells (IC50 = 5.87 μg/mL) is noteworthy because this cell line carries a BRCA1 mutation, has impaired homologous recombination repair, and presents an altered p53 context. Although the observed cytotoxicity in this p53-mutated model is compatible with the involvement of p53-independent mechanisms, the present viability data do not establish whether the response is dependent on p53 status or mediated by alternative pathways. Mitochondrial dysfunction, oxidative stress, DNA-damage accumulation, and activation of alternative apoptotic pathways may represent possible explanations. In addition, previous studies have reported that germacrene D can induce cytotoxicity in human tumor cells, including MDA-MB-231 and HCT-116 cells, through mechanisms associated with mitochondrial dysfunction and oxidative stress; however, these findings cannot be directly extrapolated to YEO-treated MDA-MB-436 cells or used to establish the mechanism of action in the present study. Therefore, these interpretations should be considered hypotheses rather than demonstrated mechanisms. Further studies measuring p53 expression and transcriptional activity, mitochondrial membrane potential, reactive oxygen species, caspase activation, DNA-damage markers, and the effects of pathway-specific inhibitors or genetic perturbations are required to determine whether YEO-induced cytotoxicity in MDA-MB-436 cells involves p53-independent signaling, mitochondrial dysfunction, oxidative stress, DNA-damage responses, or alternative apoptotic pathways.

The IC50 value measured in PBMCs was 846.40 ± 22.84 μg/mL, which was higher than the corresponding IC50 values measured in the three breast-cancer cell lines under the present experimental conditions. The resulting preliminary selectivity index (SI) values were 209.9 for MCF-7, 144.2 for MDA-MB-436, and 14.3 for MDA-MB-231. These values indicate lower susceptibility of PBMC metabolic viability to YEO than that of the tested tumor-cell models in this assay. However, PBMCs are suspension immune cells and are not equivalent to normal mammary epithelial cells. Consequently, these SI values should be interpreted as assay- and model-dependent comparative indices rather than as definitive measures of tumor selectivity, therapeutic window, or clinical safety. Differences in cell lineage, adherence, proliferation rate, metabolism, and assay response may contribute to the observed values. Evaluation using non-tumorigenic mammary epithelial cells, such as MCF-10A, and additional normal-cell models will be required to assess tissue-specific selectivity.

This result is in agreement with a study carried out in 2025 by Madrid et al., who found a selectivity index of 24.01 for *Gomortega keule* essential oil when compared with respect to breast cancer cells and normal MCF-10A cells, the selectivity being due to a ROS-mediated pro-apoptotic mechanism [18]. The authors showed that the essential oil was able to achieve such a high degree of selectivity by taking advantage of the different oxidative stress thresholds existing between cancer cells and normal cells. With regard to YEO, the fact that it contains β-caryophyllene at 15.17% is especially important since Ahmed (2025) stated that this compound acts as a dual modulator of oxidative stress by increasing reactive oxygen species (ROS) in cancer cells, while at the same time reducing ROS in normal tissues [12]. Previous studies have proposed this mechanism as a possible explanation for differential sensitivity between cancer and non-cancer cells; however, we did not investigate it in the present study. Moreover, the PBMC comparison provides only a preliminary indication of differential susceptibility and does not establish a safety profile for normal breast tissue.

The preliminary SI value of 14.3 for MDA-MB-231 was lower than the values calculated for MCF-7 and MDA-MB-436. However, because this comparison was based on PBMCs rather than normal mammary epithelial cells, it should not be interpreted as evidence of confirmed breast-cancer selectivity, a therapeutic window, or clinical safety. Previous studies of YEO-containing nanovesicles reported enhanced cytotoxicity under their specific experimental conditions [19], but those findings cannot be directly extrapolated to the present commercial YEO preparation or used to establish its safety or therapeutic index. Further studies using non-tumorigenic mammary epithelial cells, multiple normal-cell models, and validated toxicity assays are required to evaluate tissue-specific selectivity.

The use of PBMCs as a comparator is an additional limitation. PBMCs are suspension immune cells and differ substantially from normal mammary epithelial cells in lineage, morphology, proliferation, metabolism, adherence, and drug response. Therefore, the high SI values calculated relative to PBMCs should not be interpreted as a definitive measure of breast-tissue selectivity or therapeutic safety. Future studies should include non-tumorigenic mammary epithelial cells, such as MCF-10A, additional normal human-cell models, and orthogonal viability and toxicity assays.

### 2.3. Computational Results

#### 2.3.1. Deep Learning Structural Modeling and Confidence Assessment via AlphaFold 2

The pLDDT diagrams illustrate how confident the predictions are. Any areas with a high pLDDT value (usually above 70) are regarded as well-predicted and suitable for use in docking analysis, while those with lower scores (typically below 50) might point to intrinsically disordered regions or to structural uncertainty. The AlphaFold 2 models showed generally favorable pLDDT values in several structured regions of ER-α, EGFR, and PARP1 (Figure 3). These results were used only as an exploratory comparison with the corresponding experimental structures. They do not validate the crystallographic structures, the docking protocol, or the predicted ligand interactions. The docking calculations were based on the experimentally determined PDB structures.

#### 2.3.2. Gnina Molecular Docking Analysis

The Gnina docking analysis provided a preliminary exploratory screening of the identified ylang-ylang constituents against the three breast-cancer-relevant targets. Heatmaps of CNNScore (Figure 4) and CNNAffinity (Figure 5) were used to visualize the predicted pose-confidence and affinity-related scores across the compound library. Because the calculations were performed with an exhaustiveness setting of 1, the resulting scores and ligand rankings should be interpreted as preliminary, hypothesis-generating predictions rather than as definitive estimates of binding affinity or optimal binding poses.

Based on the composite score (CNNScore × CNNAffinity), the top-performing ligands for each receptor are presented in Table 3.

The present study combined GC–MS characterization, in vitro cytotoxicity testing, preliminary exploratory docking, and in silico ADMET profiling to characterize one commercial ylang-ylang essential oil preparation. The main findings were the sesquiterpene-rich chemical profile, differential sensitivity of the breast-cancer cell models to YEO, and method-dependent prioritization of individual constituents by Gnina and Glide. These results provide candidate molecules and testable mechanistic hypotheses, but they do not establish direct target inhibition, a specific mode of cell death, complex stability, synergism, or clinical efficacy [2,17].

The identification of trans-Geranylgeraniol as a top ligand for ER-α (3ERT) is particularly significant, given ER-α’s central role in hormone-dependent breast cancers. Previous studies have highlighted the cytotoxic effects of sesquiterpenes, abundant in ylang-ylang, against MCF-7 cells [2]. The strong binding of Farnesyl-decanoate to EGFR (1M17) suggests its potential in targeting aggressive breast cancer subtypes like MDA-MB-231, where EGFR overexpression is common [20]. The promising interaction of Linalool with PARP1 (4ZZZ) aligns with recent research validating Linalool’s ability to inhibit proliferation and induce apoptosis in breast cancer cells, potentially through DNA repair pathway modulation [21]. Benzyl-acetate also emerged as a strong PARP1 inhibitor, further supporting the multi-target nature of ylang-ylang components.

Although the in silico results are very promising, it is important to admit the limitations involved. Docking calculations provide only theoretical values for binding affinity and binding poses, which must be verified experimentally. Moreover, the ADMET predictions provide only an initial evaluation of the pharmacokinetic properties, and in vitro and in vivo studies are necessary to establish bioavailability, metabolism, and toxicity. Future research should therefore concentrate on synthesizing and testing these lead compounds, both separately and in combination, in order to determine their synergistic effects and therapeutic efficacy in appropriate breast cancer models.

#### 2.3.3. Docking Analysis

ER-α, widely expressed in the mammary gland and uterus, is a nuclear receptor that plays a critical role in regulating cell growth, differentiation, and proliferation in breast tissue. The overactivity of estrogen signaling through ER-α contributes to the development and progression of ER-positive breast cancers, making it a primary therapeutic target. Inhibition of ER-α disrupts estrogen-dependent transcriptional activation, leading to cell cycle arrest and apoptosis in hormone-dependent breast cancer cells. The MCF-7 cell line, an ER-positive human breast adenocarcinoma model, is the most commonly used system for evaluating antiestrogen therapies [22].

In our in silico study, the phytochemical constituents of ylang-ylang essential oil were evaluated against the ER-α receptor (PDB ID: 3ERT). The results are summarized in Table 4.

In our in silico study, δ-Cadinene, α-Cadinol, and Germacrene D were the most active molecules against ER-α, with Glide GScores of −8.278, −8.218, and −8.024 kcal/mol, respectively. These sesquiterpenes demonstrated binding affinities comparable to or exceeding those of reference antiestrogen compounds reported in the literature. Rafeeq et al. (2022) reported that phytochemical lead compounds ZINC69481841 and ZINC95486083 bound to ER-α with binding energies of −10.47 and −11.88 kcal/mol, respectively, while the reference compound tamoxifen exhibited a binding energy of −8.32 kcal/mol [22]. Notably, the Glide GScore of δ-Cadinene (−8.278 kcal/mol) approaches the binding energy of tamoxifen, suggesting that this compound may serve as a putative ER-α-interacting constituent. δ-Cadinene did not form any conventional hydrogen bonds with the amino acid residues within the active site of ERα (Figure 6A,B). Nevertheless, its favorable binding affinity may be attributed to hydrophobic interactions and van der Waals contacts within the binding pocket, which contribute to the stabilization of the ligand–receptor complex.

The strong binding affinity of δ-cadinene to ER-α is consistent with recent biological evidence. Reyes-Vidal et al. (2025) demonstrated that δ-cadinene significantly reduced cell viability and impaired invasive capacity in breast cancer models while exerting minimal effects on normal breast epithelial cells (MCF10-A), indicating selective anticancer activity [23]. Furthermore, Nguyen et al. (2022) identified δ-cadinene and γ-Muurolene as potential active compounds against MCF-7 cells through machine-learning-based screening combined with molecular docking analysis, corroborating our computational findings [24]. The presence of these sesquiterpenes as major constituents of ylang-ylang essential oil, as confirmed by GC-MS profiling, supports the hypothesis that the oil exerts its anticancer effects, at least in part, through ER-α modulation.

However, YEO is a complex mixture containing all identified constituents, and the docking score of an individual compound cannot establish that δ-cadinene is the sole or predominant mediator of the biological activity of the complete oil. Accordingly, δ-cadinene should be regarded as a putative contributor and a candidate ER-α-interacting constituent rather than as an experimentally confirmed ER-α modulator. The biological activity of YEO may reflect the combined effects of several constituents, including δ-cadinene, α-cadinol, germacrene D, γ-muurolene, β-caryophyllene, and α-humulene. These interactions may be additive, synergistic, or antagonistic, and their nature cannot be inferred from docking scores alone.

EGFR is a transmembrane tyrosine kinase receptor that is highly overexpressed in MDA-MB-231 triple-negative breast cancer cells. EGFR overexpression promotes tumor progression, angiogenesis, and metastasis through the activation of downstream signaling pathways including the RAS/MAPK and PI3K/AKT cascades. Inhibition of EGFR tyrosine kinase activity represents a validated therapeutic strategy for managing aggressive breast cancer subtypes [2].

In our in silico study, the phytochemical constituents of ylang-ylang essential oil were evaluated against the EGFR tyrosine kinase domain (PDB ID: 1M17). The results are summarized in Table 4. Benzoic acid, 2-hydroxy-, phenylmethyl ester (salicylate benzyl ester), benzyl benzoate, and 4a(2H)-Naphthalenol were the most active molecules against EGFR, with Glide GScores of −6.573, −6.262, and −6.218 kcal/mol, respectively. The prominence of benzyl ester derivatives among the top-ranking compounds is noteworthy. Aljohani et al. (2024) demonstrated that benzyl-benzoate analogs function as anti-tyrosine kinase agents targeting EGFR, with molecular docking analyses confirming their ability to bind within the ATP-binding pocket of the kinase domain [25]. The results of the present study are consistent with this finding, suggesting that the aromatic ester components of ylang-ylang essential oil may interfere with EGFR-mediated signaling in MDA-MB-231 cells.

In the active site of EGFR, benzoic acid, 2-hydroxy-, phenylmethyl ester established two hydrogen bonds with GLU 738 and THR 830 (Figure 7A,B).

Sedky et al. (2023) previously demonstrated that ylang-ylang essential oil, when formulated as pH-responsive niosomes, exhibited significant cytotoxic activity against MDA-MB-231 cells with an IC_50_ of 18.39 µg/mL [2]. The co-delivery of ylang-ylang oil with oxaliplatin in nanovesicles further enhanced cytotoxicity, achieving an IC_50_ of 0.0002 µg/mL through induction of the intrinsic apoptotic pathway involving upregulation of p53, Bax, and Caspase-7 [2]. The present molecular docking results provide a structural basis for these biological effects, indicating that benzyl benzoate and related aromatic esters may contribute to EGFR inhibition as a mechanism underlying the anticancer activity of ylang-ylang essential oil.

Poly (ADP-ribose) polymerase 1 (PARP1) is a multifunctional enzyme that plays a central role in the DNA damage response, facilitating the repair of single-stranded DNA breaks via the base excision repair (BER) pathway. Its therapeutic relevance is particularly pronounced in breast cancer associated with BRCA1 or BRCA2 mutations, where compromised homologous recombination repair (HRR) creates a synthetic lethal dependency on PARP1-mediated repair [26]. The MDA-MB-436 cell line harbors a BRCA1 mutation, rendering it exquisitely sensitive to PARP1 inhibition. FDA-approved PARP inhibitors such as Olaparib, Rucaparib, and Talazoparib have demonstrated significant clinical efficacy in BRCA-mutated breast cancers, with Talazoparib achieving an IC_50_ of approximately 1 nM in MDA-MB-436 cells [27].

In our in silico study, the phytochemical constituents of ylang-ylang essential oil were evaluated against PARP1 (PDB ID: 4ZZZ). The results are summarized in Table 4. Benzyl benzoate, methyl benzoate, and 4a(2H)-Naphthalenol were the most active molecules against PARP1, with Glide GScores of −8.058, −7.552, and −7.139 kcal/mol, respectively. These docking scores are notably comparable to those reported for established PARP1 inhibitors. Twala et al. (2025) reported that Olaparib, Rucaparib, and Talazoparib achieved docking scores between −9.0 and −9.3 kcal/mol against the PARP1 catalytic domain, while quinazolinone-based inhibitors reached −9.2 kcal/mol [26]. Although the Glide GScores of benzyl benzoate (−8.058 kcal/mol) and methyl benzoate (−7.552 kcal/mol) are somewhat lower than those of the FDA-approved PARP inhibitors, they remain within a range suggestive of meaningful binding interactions at the PARP1 active site.

Benzyl benzoate established two hydrogen bonds with residues GLY863 and SER 904 and one Pi–Pi stacking bond with residue TYR 907 in the active site of PARP1 (Figure 8A,B).

The biological significance of these findings is underscored by the synthetic lethality paradigm. Yan et al. (2023) demonstrated that molecular dynamics simulations reveal critical van der Waals interactions between PARP1 inhibitors and the catalytic pocket residues, emphasizing the importance of lipophilic moieties in achieving effective PARP1 binding [27]. The aromatic ester structures of benzyl benzoate and methyl benzoate, which feature lipophilic benzyl groups, may facilitate similar interactions within the PARP1 nicotinamide-binding pocket. Furthermore, Keung et al. (2020) reported that MDA-MB-436 cells showed the most pronounced response to PARP inhibition among breast cancer cell lines, with Talazoparib achieving an IC_50_ of approximately 0.13 µM, validating this cell line as a sensitive model for PARP-targeted therapies [28].

The analysis of the compounds carried out using both Gnina and Glide shows that the constituents of ylang-ylang could interact with a number of targets relevant to breast cancer, although the order in which the most active molecules appeared differed depending on the docking method. Gnina ranked the ligands on the basis of the CNNScore × CNNAffinity combined metric, while Glide ranked the compounds by means of its empirical GScore. Thus, those compounds that were identified by both methods should be regarded as higher-priority candidates than those found by just one of the two scoring systems.

With respect to ER-α (PDB ID: 3ERT), Gnina found trans-geranylgeraniol, farnesyl-decanoate and cis-β-copaene to be the best compounds, while Glide ranked δ-cadinene, α-cadinol and germacrene D as the most favorable, the GScores being −8.278, −8.218 and −8.024 kcal/mol, respectively. For EGFR (PDB ID: 1M17), farnesyl-decanoate was the top compound identified by Gnina, whereas Glide selected benzyl salicylate, benzyl benzoate and 4a(2H)-naphthalenol, with GScores of −6.573, −6.262 and −6.218 kcal/mol. In the case of PARP1 (PDB ID: 4ZZZ), Gnina chose linalool and α-muurolene as its top candidates, while Glide determined that benzyl benzoate, methyl benzoate and 4a(2H)-naphthalenol were the strongest binders. It is notable that benzyl benzoate showed favorable predicted interactions with both EGFR and PARP1 and that α-cadinol had strong Glide scores for ER-α and PARP1 as well. δ-Cadinene also had the highest Glide affinity for ER-α and, at the same time, maintained a moderate predicted binding to both EGFR and PARP1.

Nevertheless, these rankings should be interpreted as prioritization of candidate individual constituents and not as evidence of their relative contribution to the activity of the complete YEO. The mixture contains compounds with different abundances, physicochemical properties, cellular availability, and potential targets. Docking was performed separately for each ligand and therefore does not capture simultaneous interactions, concentration-dependent effects, or additive, synergistic, or antagonistic interactions among constituents. Furthermore, a further limitation of the present study is the absence of Molecular Dynamics simulations. Static docking does not account for time-dependent protein flexibility, solvent effects, ligand dissociation, or the persistence of the predicted interactions. Therefore, the docking results cannot establish complex stability, direct target engagement, target inhibition, or biological significance. The predicted interactions should consequently be regarded as preliminary structural hypotheses.

These results support a potential polypharmacological mechanism involving hormone signaling, receptor tyrosine-kinase pathways, and DNA-damage repair. The activity of sesquiterpenes such as δ-cadinene, α-cadinol, germacrene D, and α-humulene is consistent with previous reports of anticancer effects in breast-cancer models [29,30]. However, docking cannot prove synergism. The proposed multi-target effect should therefore be validated through biochemical assays, molecular-dynamics simulations, and combination studies using Bliss, Loewe, or Chou–Talalay models.

Accordingly, the present findings support a multi-component and multi-target hypothesis for YEO activity, but they do not establish that δ-cadinene alone is responsible for ER-α-related effects. Purified-compound testing, reconstructed mixtures reflecting the measured composition, and formal combination analyses will be required to distinguish additive, synergistic, and antagonistic effects.

#### 2.3.4. Physicochemical Properties and Drug-Likeness

The in silico ADMET profiling of the all ylang-ylang essential oil constituents revealed distinct physicochemical profiles aligned with their molecular classes. As summarized in Table 5, the compounds evaluated span a range of chemical families, including monoterpenes (e.g., β-Myrcene, Linalool, and Geraniol), sesquiterpenes (e.g., δ-Cadinene, α-Cadinol, Caryophyllene, and Germacrene D), and aromatic esters (e.g., Benzyl Benzoate, Methyl Benzoate, and Benzoic acid, 2-hydroxy-, phenylmethyl ester).

The molecular weights of the identified compounds ranged from 122.16 g/mol (3-Methoxytoluene) to 376.60 g/mol ((E,E)-3,7,11-Trimethyl-2,6,10-dodecatrienyl decanoate). The QPlogPo/w values, which estimate lipophilicity, varied significantly across the dataset, from 2.119 for Methyl Benzoate to 8.244 for (E,E)-3,7,11-Trimethyl-2,6,10-dodecatrienyl decanoate. The highly lipophilic sesquiterpenes, such as δ-Cadinene (5.356) and γ-Muurolene (5.585), exhibited substantial partition coefficients, characteristic of volatile essential oil constituents that facilitate rapid absorption across biological membranes but may present challenges regarding aqueous solubility.

A critical aspect of drug-likeness evaluation is the assessment of hydrogen bonding capacity and rule violations. The evaluated compounds demonstrated hydrogen bond donor counts ranging from 0.0 to 1.0 and acceptor counts ranging from 0.0 to 2.0. Notably, 15 of the 30 compounds exhibited zero violations of Lipinski’s Rule of Five and Veber’s Rule of Three. Compounds such as Benzyl Benzoate, Methyl Benzoate, and Benzoic acid, 2-hydroxy-, phenylmethyl ester showed no violations, indicating their strong potential suitability as orally active pharmaceutical agents. Conversely, highly lipophilic sesquiterpenes like δ-Cadinene and α-Humulene exhibited one violation of the Rule of Five, primarily due to their high lipophilicity (QPlogPo/w > 5.0).

The pharmacokinetic predictions generated by QikProp provide crucial insights into the potential bioavailability and tissue distribution of the ylang-ylang constituents. Aqueous solubility (QPlogS) is a fundamental parameter influencing oral absorption. The evaluated compounds exhibited a range of solubility profiles, with highly lipophilic sesquiterpenes such as δ-Cadinene (−6.338) and α-Cadinol (−4.307) demonstrating lower aqueous solubility compared to smaller, more polar monoterpenes and esters like methyl benzoate (−1.844) and benzyl acetate (−2.065).

Cell permeability, estimated via Caco-2 (QPPCaco) and MDCK (QPPMDCK) models, is indicative of the compounds’ ability to traverse biological barriers. Interestingly, many of the highly lipophilic sesquiterpenes (e.g., δ-Cadinene, γ-Muurolene, and Caryophyllene) exhibited maximum predicted permeability values (QPPCaco = 9906.038 nm/s), suggesting rapid and efficient absorption. The brain/blood partition coefficient (QPlogBB) predictions further elucidate the potential for central nervous system penetration, with most compounds exhibiting values below 1.0, indicating limited brain penetration, which may be advantageous for minimizing neurological side effects in systemic therapeutics.

Toxicity predictions focused on cardiotoxicity via the assessment of hERG K+ channel inhibition. The QPlogHERG values for the ylang-ylang constituents ranged from −2.943 to −5.771. Values below −5.0 are generally considered indicative of a low risk for hERG-mediated cardiotoxicity. Notably, compounds such as (E,E)-3,7,11-Trimethyl-2,6,10-dodecatrienyl decanoate (−5.771), benzoic acid, 2-hydroxy-, phenylmethyl ester (−5.557), and benzyl benzoate (−5.653) exhibited highly favorable profiles, suggesting a significantly reduced propensity for inducing arrhythmias. In contrast, highly lipophilic compounds like α-Humulene (−2.943) and trans-Geranylgeraniol (−4.968) showed values closer to the threshold, warranting careful monitoring in future in vivo studies.

The integration of these ADMET predictions with the molecular docking results supports the prioritization of specific ylang-ylang constituents for further investigation. For instance, benzyl benzoate, which demonstrated strong predicted binding to both EGFR and PARP1, also exhibited a balanced pharmacokinetic profile with a QPlogPo/w of 3.571, zero hydrogen bond donors, two acceptors, and no violations of the Rule of Five. Similarly, δ-Cadinene, which showed a favorable predicted docking score against ER-α, displayed favorable lipophilicity and no cardiotoxicity alerts despite one rule violation due to high logP. These in silico properties do not establish therapeutic efficacy or confirm ER-α modulation.

These in silico findings suggest that selected ylang-ylang constituents may warrant further investigation because of their predicted target interactions and preliminary pharmacokinetic profiles. However, the present data do not establish anticancer efficacy, therapeutic effectiveness, or clinical potential. Further biochemical, mechanistic, pharmacokinetic, and in vivo studies are required to evaluate their potential anticancer activity and translational relevance.

## 3. Materials and Methods

### 3.1. Plant Materials

An organic ylang-ylang essential oil derived from the flowers of *Cananga odorata* (Annonaceae) was purchased from Puressentiel (Brussels, Belgium/France). According to the manufacturer’s publicly available product information, the oil is 100% pure and natural, produced from organic agriculture, and classified as an Essential Oil Botanically and Biochemically Defined (EOBBD) product. The producing organ is the flower, and the oil is obtained by complete distillation. The manufacturer identifies benzyl acetate, linalool, and germacrene D as characteristic chemotype constituents. Ylang-ylang essential oil is obtained by steam distillation of the flowers; the oil is therefore a neat, anhydrous mixture of volatile constituents, and it was used as supplied, without further purification. Bacterial endotoxins (lipopolysaccharides) are heat-stable amphiphilic macromolecules (monomers of 10–20 kDa that self-assemble into aggregates of up to about 1000 kDa) [31] and are non-volatile; they are therefore not carried into the distillate, which is why distillation is the reference process for producing Water for Injections that complies with the pharmacopeial bacterial-endotoxin limit [32]. The endotoxin content of the oil was not measured directly; the Limulus amebocyte lysate (LAL) assay is prone to interference from turbid or colored samples [31], as are aqueous dispersions of essential oils, and the absence of a direct endotoxin assay is acknowledged as a limitation of the present study.

### 3.2. Essential Oil Compounds Identification

To determine the chemical composition of YEO, we used gas chromatography–mass spectrometry (GC–MS), a method that separates and identifies compounds that readily evaporate. The tests were done on a Shimadzu GCMS-TQ8040 NX Triple Quadrupole GC–MS system (Shimadzu, Tokyo, Japan), using a non-polar RTxi-5 Sil MS capillary column (30 m long, 0.25 mm wide, and 0.25 μm thick). Before testing, the essential oils were carefully mixed with n-hexane (10 parts oil to 100 parts solvent). A small 1 μL sample of this mix was injected in split mode (split ratio 1:20). Helium gas, chosen because it does not react, flowed steadily at 1.0 mL per minute, while the injector was kept at 250 °C. The GC oven followed a careful temperature plan: starting at 50 °C for 2 min, then increasing by 5 °C per minute to 160 °C (held for 2 min), then rising at the same rate to 280 °C (held for 2 min), with the whole test taking about 50 min. The mass spectrometer worked in electron ionization (EI) mode at 70 eV, with the ion source at 200 °C and the interface at 280 °C. Data were recorded in full-scan mode over the *m*/*z* range 40–650. To confirm the compounds, the measured Kovats retention indices were compared with reference values from the NIST 98 and Adams databases [33,34].

### 3.3. Cytotoxicity Against Breast Cancer Cell Lines

Three human breast cancer cell lines were used: the estrogen-receptor-positive (MCF-7) and the estrogen-receptor-negative (MDA-MB-231 and MDA-MB-436). The cell lines were obtained from the American Type Culture Collection (ATCC, Manassas, VA, USA; MCF-7, HTB-22; MDA-MB-231, HTB-26; MDA-MB-436, HTB-130). Cells were grown at 37 °C in Dulbecco’s modified Eagle’s medium (DMEM; Sigma-Aldrich, St. Louis, MO, USA) with 10% fetal bovine serum (FBS; Sigma-Aldrich) and 50 μg/mL of gentamicin (Sigma-Aldrich) in a humidified 5% CO2 environment. Cultures were kept in 25 cm^2^ tissue culture flasks. Cells actively growing were selected for viability testing.

To test the ability of YEO to inhibit cancer cell growth, the MTT assay was performed according to standard methods [35,36]. YEO was dissolved in DMSO (Sigma-Aldrich) and serially diluted in culture medium to final concentrations of 0.375, 0.75, 1.5, 3, 10, 30, 75 and 100 μg/mL (final DMSO concentration 0.1%, *v*/*v*). Cisplatin (Sigma-Aldrich) was used as a positive control at 0.01, 0.1, 1, 5, 10, 25 and 50 µg/mL. Cells were treated with YEO or cisplatin for 72 h; control wells got medium with 0.1% DMSO. After treatment, 200 μL of culture medium and 20 μL of MTT reagent (Sigma-Aldrich; 5 mg/mL in phosphate-buffered saline) were added to each well, and the plate was incubated at 37 °C for 4 h. The medium was then removed, and 100 µL of DMSO was added to dissolve the formazan crystals. Absorbance was measured at 540 nm using an HT Multi-Detection Microplate Reader (Bio-Tek, Winooski, VT, USA) to check cell viability [36]. The effect of the treatments on cell viability was calculated using the following equation:

(1) $$\mathrm{Cell}\;\mathrm{viability}\;\left(\%\right)=100-\left[\left(\left(A_{0}-A_{t}\right)/A_{0}\right)\times 100\right]$$

A_0_ is the absorbance of cells treated with 0.1% DMSO medium, and A_t_ is the absorbance of cells treated with YEO or cisplatin at the different concentrations. Negative controls received 0.1% DMSO in the medium. Peripheral blood mononuclear cells (PBMCs) were isolated from peripheral blood of healthy adult volunteers, who gave written informed consent, using Ficoll–Hypaque density centrifugation, following the manufacturer’s instructions (Capricorn Scientific, Ebsdorfergrund, Germany). The Research Ethics Committee reviewed and approved all studies involving human participants (03/22-LAPABE-10 and 4 March 2022) before experimentation. PBMCs were included as a preliminary non-tumoral human-cell reference model, and the cytotoxic effects of YEO and cisplatin on PBMCs were evaluated under the same conditions and concentrations as those used for tumor cells. Because PBMCs are suspension immune cells and are biologically distinct from normal mammary epithelial cells, they were not considered a direct normal-breast-tissue control.

### 3.4. Computational Methodology

#### 3.4.1. Data Retrieval and Ligand Preparation

Different phytoconstituents, comprising monoterpenes, sesquiterpenes, oxygenated derivatives, and esters, were recorded. The canonical SMILES strings and three-dimensional structural data for each identified ligand were obtained from the PubChem database.

The ligands were prepared for molecular docking by carrying out molecular geometry optimization and energy minimization with the help of the RDKit chemoinformatics library (version 2023.09.1), which is embedded in Python 3.12.3 [37]. The hydrogens were added explicitly to each molecule and the 3D coordinates were generated by means of the Experimental Torsion Knowledge Distance Geometry (ETKDG) method [38]. Then, geometry optimization was carried out using the Merck Molecular Force Field (MMFF94s) so that each ligand would take on its lowest-energy, most stable conformation. The optimized structures were exported in both Protein Data Bank (PDB) and Structure-Data File (SDF) formats.

#### 3.4.2. Protein Target Selection and Structural Retrieval

For this study, three important macromolecular targets that play a role in the development and proliferation of breast cancer were chosen: ER-α (PDB ID: 3ERT), which is the main therapeutic receptor for hormone-dependent breast cancer cell lines such as MCF-7 [39]; EGFR (PDB ID: 1M17), a tyrosine kinase receptor that is often overexpressed in aggressive, triple-negative breast cancer cell lines such as MDA-MB-231 [40]; and PARP1 (PDB ID: 4ZZZ), a key enzyme involved in the repair of DNA damage and thus serving as the primary target for assessing activity against MDA-MB-436 cells [41].

The high-resolution 3D crystal structures of these receptors were obtained directly from the Protein Data Bank (PDB). Before carrying out the docking calculations, the receptor files were pre-processed by removing all heteroatoms, the crystallographic water molecules, and the native ligands that had co-crystallized with the receptors. Polar hydrogen atoms and Kollman charges were then added using the utilities in AutoDockTools (accessed on 23 July 2026), after which the cleaned receptor files were converted into PDBQT format where necessary.

#### 3.4.3. Deep Learning Structural Modeling and Confidence Assessment via AlphaFold 2

AlphaFold 2 models were generated only as an exploratory structural comparison with the experimentally determined receptor structures used for docking. The pLDDT values were interpreted as confidence estimates for the predicted AlphaFold models and were not used to assess or validate the quality of the crystallographic PDB structures. AlphaFold 2 employs a complex deep learning architecture to predict protein structures with atomic accuracy, and the pLDDT metric gives a confidence value on a per-residue basis ranging from 0 to 100:

pLDDT > 90: indicates very high confidence, suitable for atomic-level interaction analysis.

70 < pLDDT ≤ 90: indicates a confident prediction and is highly reliable for molecular docking.

50 < pLDDT ≤ 70: represents low confidence, indicating potential conformational flexibility or dynamic behavior.

pLDDT ≤ 50: suggests low confidence or intrinsically disordered regions.

The per-residue pLDDT profiles for each target receptor were obtained and displayed in order to ensure that the ligand-binding domains had high structural certainty before docking was carried out [42].

#### 3.4.4. AI-Based Molecular Docking via Gnina 1.0

Molecular docking was performed with Gnina (version 1.0), an advanced AI-based docking program that uses Convolutional Neural Networks (CNNs) as scoring functions to improve pose-prediction accuracy and binding-affinity estimation [43]. Unlike conventional empirical scoring functions, Gnina assesses atomic density grids by means of deep learning models that have been trained on structural databases (for example, CrossDocked2020) [44].

For each receptor, a cubic grid box of dimensions 15 Å × 15 Å × 15 Å was centered on the active binding site coordinates of the co-crystallized native ligand:

(2) $$\mathrm{ER}-\alpha \;(3\mathrm{ERT}):\;x=31.575,\;y=-1.590,\;z=25.599\;EGFR\left(1M17\right):x=22.014,\;y=0.253,\;z=52.794\;\mathrm{PARP}1\;(4\mathrm{ZZZ}):\;x=55.810,\;y=0.156,\;z=22.521$$

The docking calculations were carried out using an exhaustiveness setting of 1 as an initial, computationally economical screening condition. This low setting provides limited conformational sampling and was not intended to establish that the predicted poses represented the global optimum. Accordingly, the Gnina results were interpreted as preliminary, hypothesis-generating predictions. For each ligand–receptor pair, the binding pose with the highest score was chosen according to three main criteria:

Vina Affinity (kcal/mol): the empirical binding free energy calculated via the AutoDock Vina scoring function. CNNScore (0.0 to 1.0): the AI-derived confidence score indicating the probability that the predicted binding pose is correct. CNNAffinity: the CNN-predicted binding affinity, providing a robust correlation with experimental inhibition constants.

To rank the ligands comprehensively by balancing structural pose reliability and predicted binding strength, a composite score was formulated as:

(3) Composite Score=CNNScore×CNNAffinity

### 3.5. Molecular Docking

#### 3.5.1. Ligand Preparation

The molecular docking workflow was initiated by sourcing the targeted phytochemicals from the essential oils of *Cananga odorata* (ylang-ylang), identified by GC-MS analysis. The compounds were acquired from the PubChem database in SDF file format. Subsequent ligand preparation was conducted using the LigPrep module within the Maestro platform (Schrödinger Suite, version 11.5). Each compound underwent geometric refinement employing the OPLS3 force field, with ionization states calibrated to reflect physiological conditions (pH 7.0 ± 2.0). To ensure coverage of stereochemical variations, the system was configured to generate up to 32 stereoisomeric forms per compound [45,46].

#### 3.5.2. Protein Preparation

Protein targets integral to the docking investigation were selected based on their biological relevance to the targeted breast cancer cell lines and retrieved from the RCSB Protein Data Bank (www.rcsb.org, accessed on 23 July 2026). ER-α (PDB ID: 3ERT) was selected as the most commonly used target for ER-positive breast cancer MCF-7 cells. EGFR (PDB ID: 1M17) was chosen for the highly overexpressed receptor in MDA-MB-231 triple-negative breast cancer cells. Poly (ADP-ribose) polymerase 1 (PARP1) (PDB ID: 4ZZZ) was identified as the most relevant target for BRCA1-deficient MDA-MB-436 cells. Before performing the docking, the protein structures were prepared through a series of refinement steps, including the incorporation of hydrogen atoms, adjustment of bond orders, elimination of crystallographic water molecules, and optimization of hydrogen-bonding interactions. To improve docking accuracy, receptor charge states were adjusted, and the entire protein structures were energy-minimized using the OPLS3 force field to ensure conformational stability [47].

#### 3.5.3. Glide Standard Precision (SP) Ligand Docking

Ligand docking was carried out using the Standard Precision (SP) protocol available in the Glide module of Schrödinger’s Maestro platform (version 11.5). To enhance the reliability of the results, penalties were applied to disfavor energetically unfavorable amide orientations, and the van der Waals radii of ligand atoms were reduced to 80% of their default values. Additionally, a partial atomic charge cutoff of 0.15 was set to improve the modeling of noncovalent interactions. Docking poses were evaluated through the Glide scoring algorithm, which integrates binding strength with spatial compatibility. For each compound, the pose exhibiting the lowest Glide score—indicative of the most stable and favorable interaction—was selected as the best binding conformation [48].

#### 3.5.4. Docking Protocol Validation

To validate the molecular docking protocol, redocking experiments were performed using the crystallographic ligands co-complexed with the three target proteins employed in this study [49]. The crystal structures of Erα (PDB ID: 3ERT), EGFR (PDB ID: 1M17), and PARP1 (PDB ID: 4ZZZ) were downloaded from the RCSB Protein Data Bank. The corresponding crystallographic ligands were identified as OHT in 3ERT, AQ4 in 1M17, and FSU in 4ZZZ.

The redocking protocol reproduced the crystallographic ligand poses with RMSD values of 1.976 Å for ERα (PDB ID: 3ERT; ligand OHT), 1.542 Å for EGFR (PDB ID: 1M17; ligand AQ4), and 0.749 Å for PARP1 (PDB ID: 4ZZZ; ligand FSU). All three best poses exhibited RMSD values below 2 Å, supporting the geometric reliability of the docking protocol.

### 3.6. ADMET Prediction and Drug-Likeness Evaluation

The pharmacokinetic properties, toxicity profiles, and drug-likeness of the selected ylang-ylang constituents were evaluated using the QikProp module integrated within the Schrödinger Maestro platform. The computational predictions encompassed a comprehensive panel of pharmacokinetic descriptors, including the octanol/water partition coefficient (QPlogPo/w), aqueous solubility (QPlogS), apparent Caco-2 cell permeability (QPPCaco), apparent MDCK cell permeability (QPPMDCK), brain/blood partition coefficient (QPlogBB), and predicted binding to human serum albumin (QPlogKhsa). Furthermore, the evaluation incorporated predictions of human oral absorption and assessed potential cardiotoxicity by predicting hERG K+ channel blockage (QPlogHERG). To determine the drug-likeness of the phytochemicals, the compounds were evaluated against Lipinski’s Rule of Five and Veber’s Rule of Three, which assess the numbers of hydrogen-bond donors and acceptors and violations of established drug-likeness criteria [49].

### 3.7. Statistical Analysis

Cytotoxicity data are expressed as the mean ± standard deviation (SD) of three independent experiments (*n* = 3), each performed with three technical replicate wells per con-centration; cell viability was expressed as a percentage of the vehicle control (0.1% DMSO). Concentration–response curves were fitted by nonlinear least-squares regression using the “[inhibitor] vs. normalized response–variable slope” model, Y = 100/(1 + (IC50/X) HillSlope), in GraphPad Prism 8.01 (GraphPad Software, San Diego, CA, USA), and IC50 values were obtained from the fitted curves. Differences in IC50 between YEO and cisplatin for each cell type, and between cell types for each treatment, were tested with the extra sum-of-squares F-test on log IC50 (null hypothesis: a single log IC50 shared by the two data sets, with Hill slopes fitted separately); pairwise comparisons among the four cell types were adjusted with the Bonferroni correction. For the concentration–response data (in the section ’Cytotoxicity against Breast Cancer Cell Lines), a two-way analysis of variance (ANOVA; factors: cell type and concentration) was performed separately for YEO and cisplatin, followed by Dunnett’s multiple-comparison test comparing each breast cancer cell line with PBMCs at the same concentration, using the pooled residual error of the ANOVA (64 degrees of freedom). The ANOVA, Dunnett and F-tests were computed in Python 3 with the SciPy library (version 1.17) from the replicate data under-lying Figure 2. Differences were considered statistically significant at *p* < 0.05.

## 4. Conclusions

This study integrated GC–MS chemical profiling, in vitro cytotoxicity assays, experimental-structure-based receptor preparation, exploratory AlphaFold 2 structural comparison, Gnina screening, Glide docking, and in silico ADMET analysis to investigate the potential anticancer activity of ylang-ylang essential oil. The in vitro experiments evaluated the effects of ylang-ylang essential oil on ER-positive MCF-7 cells and triple-negative MDA-MB-231 and MDA-MB-436 cells, while peripheral blood mononuclear cells (PBMCs) were included to provide an initial indication of cytotoxic selectivity toward non-tumoral cells. Thus, the computational analyses were interpreted in the context of experimentally observed cellular activity rather than as an independent prediction alone. The in vitro results demonstrated the cellular anticancer activity of the essential oil across breast-cancer models, while the computational results indicate that its constituents may interact with three complementary cancer-relevant targets: ER-α, EGFR, and PARP1. However, the identity of the top-ranked compound depended on the docking methodology. Gnina prioritized trans-geranylgeraniol for ER-α, farnesyl-decanoate for EGFR, and linalool for PARP1 when affinity was combined with CNN-based pose confidence. Glide, in contrast, highlighted δ-cadinene, benzyl salicylate, and benzyl benzoate as the best-scoring compounds against ER-α, EGFR, and PARP1, respectively.

The comparison of both approaches identified a broader set of chemically diverse candidates with complementary target profiles. Benzyl benzoate and α-cadinol showed particularly favorable multi-target Glide scores, while δ-cadinene displayed the strongest predicted ER-α binding. These results are consistent with the hypothesis that ylang-ylang essential oil may exert its biological effects through polypharmacological activity involving hormone signaling, growth-factor pathways, and DNA-repair mechanisms. The observed in vitro cytotoxicity of the essential oil in breast-cancer models, considered together with the initial selectivity assessment in PBMCs and previous reports on the anticancer properties of selected sesquiterpenes and aromatic esters, provides additional support for the biological relevance of this hypothesis. However, the in vitro findings establish cellular activity and selectivity trends, not yet direct engagement of ER-α, EGFR, or PARP1. Nevertheless, the present findings remain predictive. Docking scores are not direct measurements of binding free energy, and differences between Gnina and Glide demonstrate that ligand ranking is model-dependent. In addition, the essential oil is a complex mixture whose activity depends on constituent abundance, absorption, metabolism, cellular uptake, and possible interactions between compounds. The docking analysis provided a preliminary prioritization of ylang-ylang constituents and generated testable hypotheses regarding their potential interactions with ER-α, EGFR, and PARP1. However, as Molecular Dynamics simulations and direct biochemical target-validation assays were not performed in the present study, the stability and biological significance of the predicted complexes remain to be established. Consequently, the data support candidate prioritization and mechanistic hypothesis generation, but they do not yet establish target inhibition, therapeutic efficacy, clinical efficacy, or synergistic action. Further Molecular Dynamics simulations and experimental validation will be necessary to confirm these predictions in future studies.

## Figures and Tables

**Figure 1:**
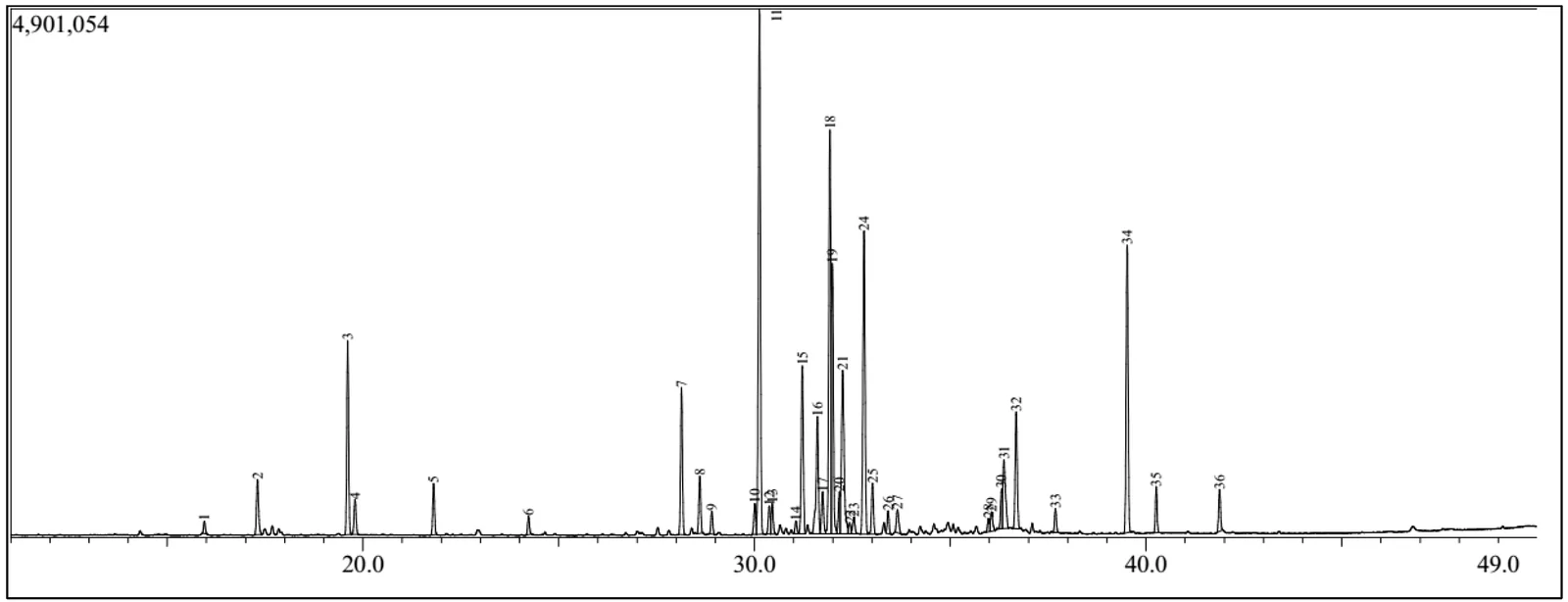
GC–MS chromatogram of the compounds identified in YEO.

**Figure 2:**
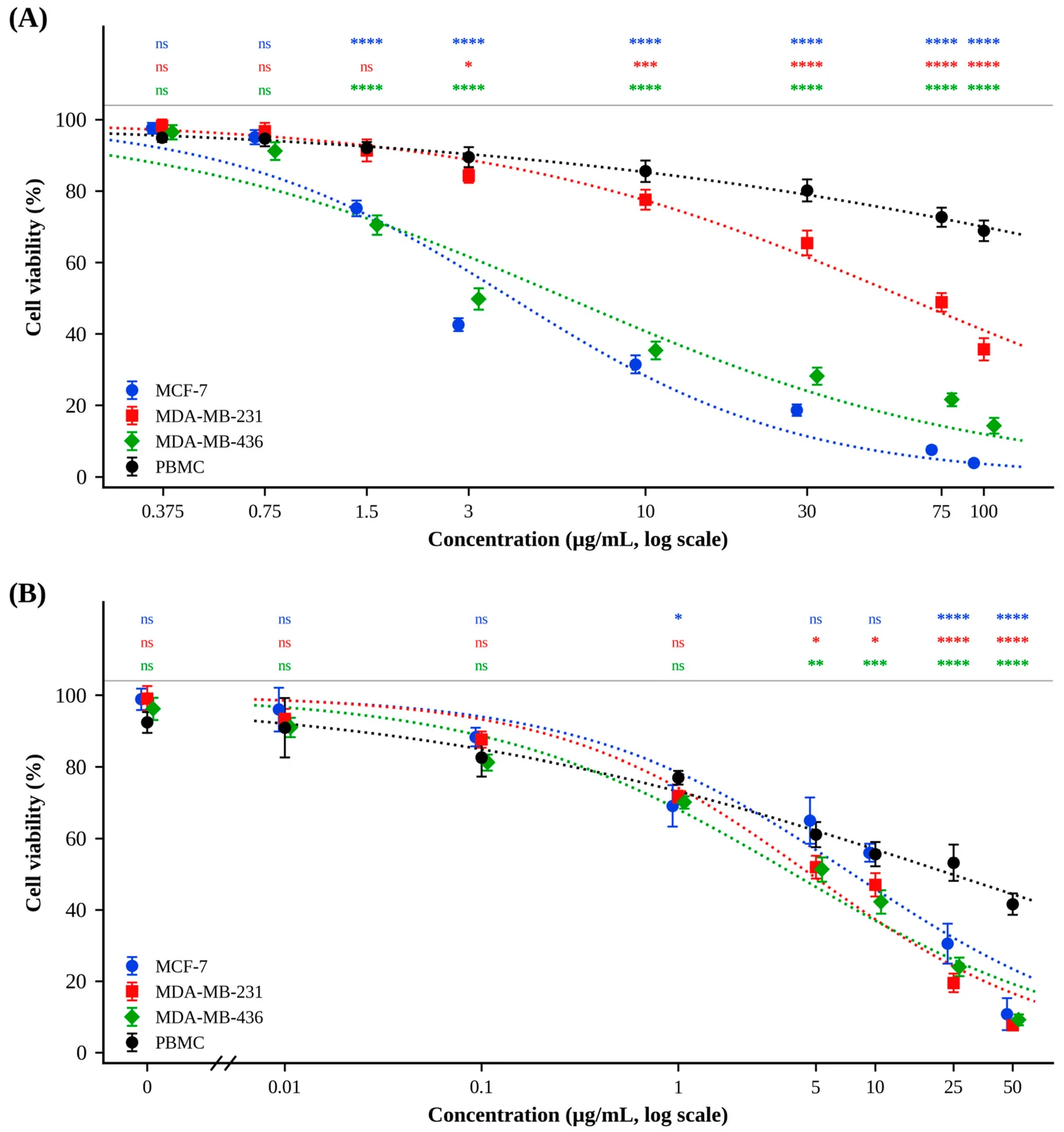
Concentration-dependent effects of YEO (**A**) and cisplatin (positive control) (**B**) on the viability of MCF-7, MDA-MB-231 and MDA-MB-436 breast cancer cells and of PBMCs after 72 h of treatment (MTT assay). Data are presented as the mean ± SD of three independent experiments (n = 3; biological replicates), each performed in technical triplicate, and are expressed as a percentage of the vehicle control (0.1% DMSO). Dotted lines show the nonlinear regression fits ([inhibitor] vs. normalized response, variable slope) used to derive the IC_50_ values. Concentrations are plotted on a logarithmic scale; in (**B**), “0” denotes the vehicle-treated control. The symbols above each panel give the significance of the difference between each cancer cell line (color-coded) and PBMCs at the same concentration (two-way ANOVA followed by Dunnett’s multiple-comparison test): ns, not significant (*p* ≥ 0.05); * *p* < 0.05; ** *p* < 0.01; *** *p* < 0.001; **** *p* < 0.0001.

**Figure 3:**
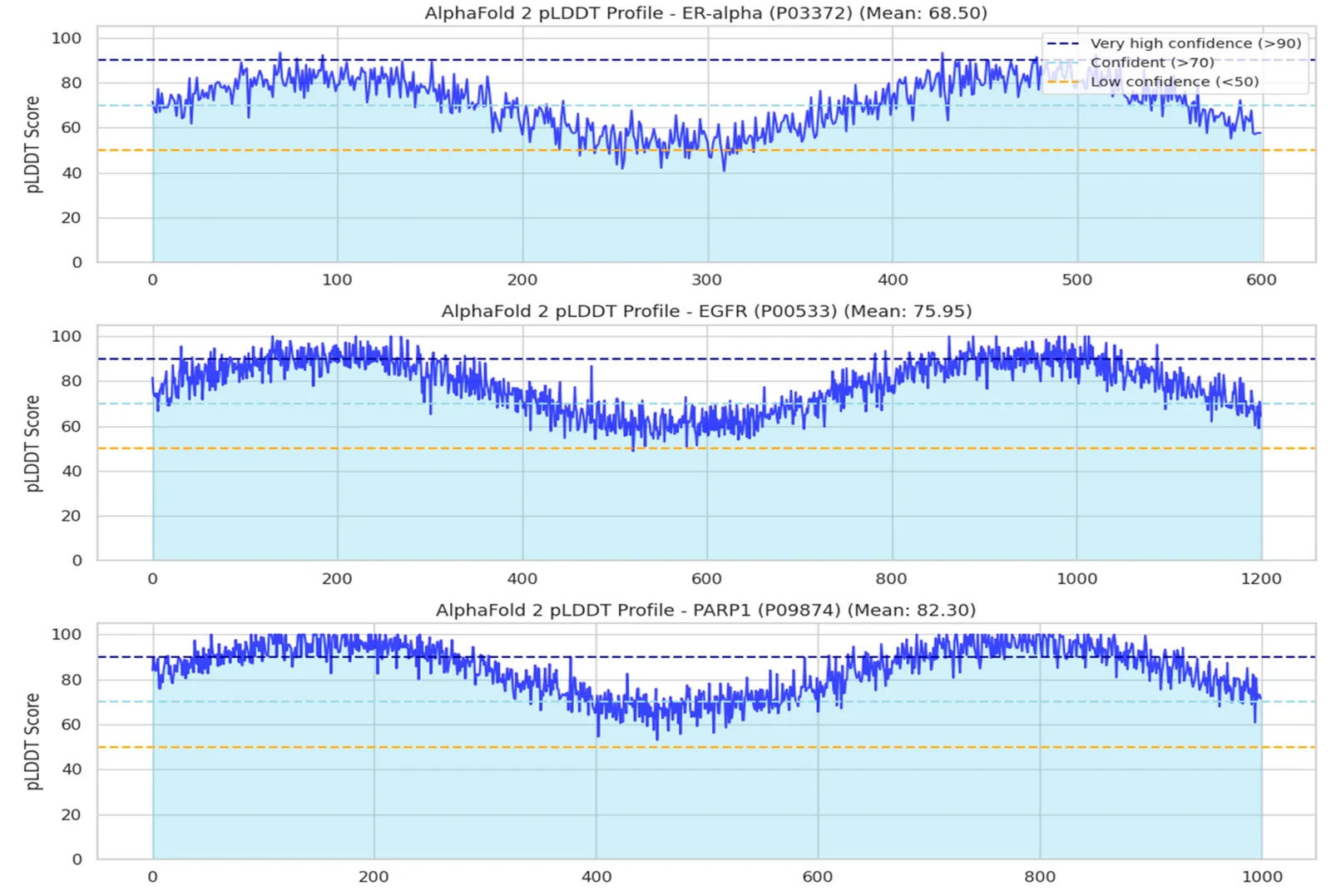
The pLDDT profiles for ER-α, EGFR, and PARP1.

**Figure 4:**
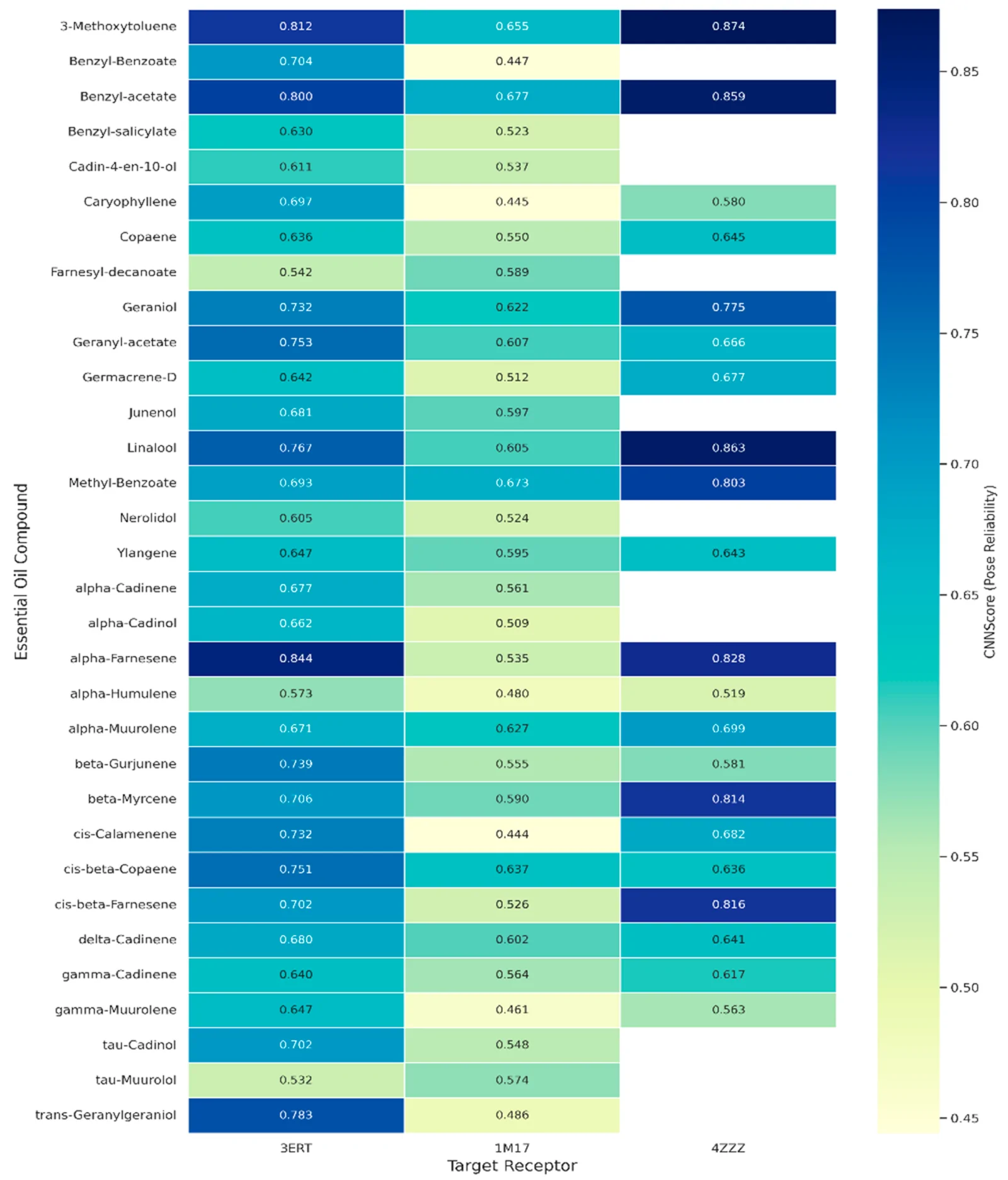
Pose reliability (CNNscore) for ylang-ylang compounds.

**Figure 5:**
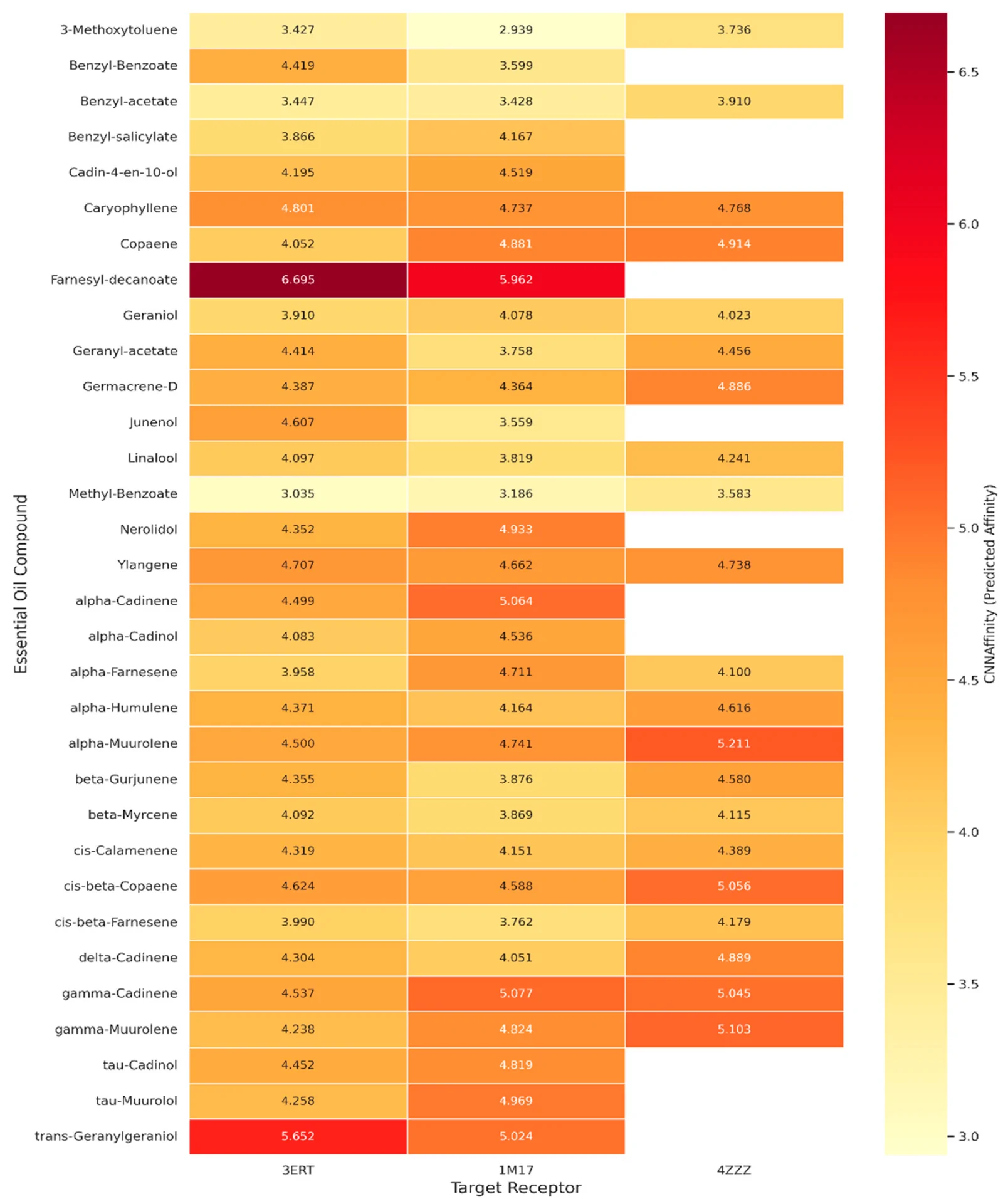
Predicted binding affinity (CNNaffinity) for ylang-ylang compounds.

**Figure 6:**
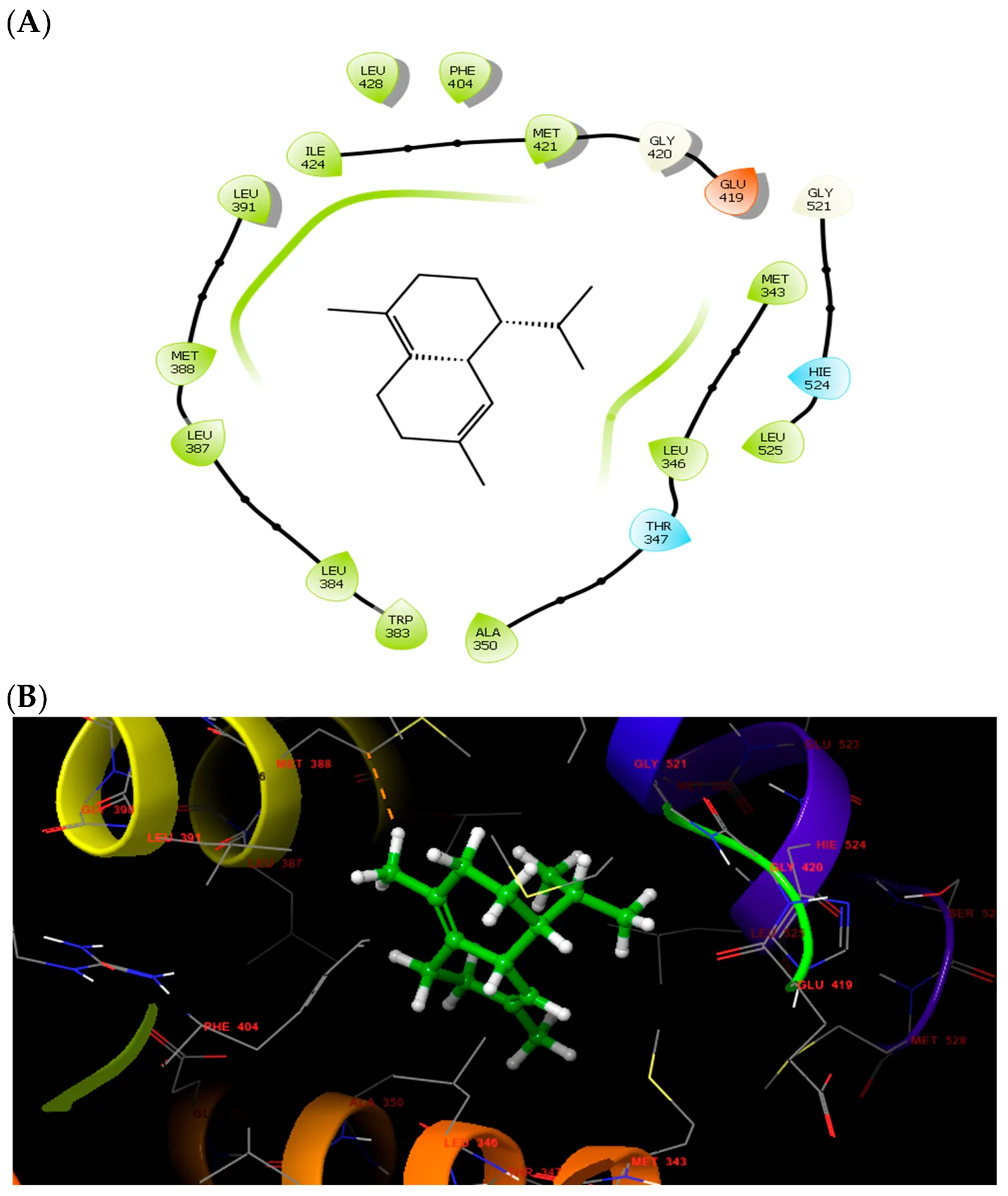
2D (**A**) and 3D (**B**) interaction of δ-cadinene in the active site of ER-α (PDB ID: 3ERT).

**Figure 7:**
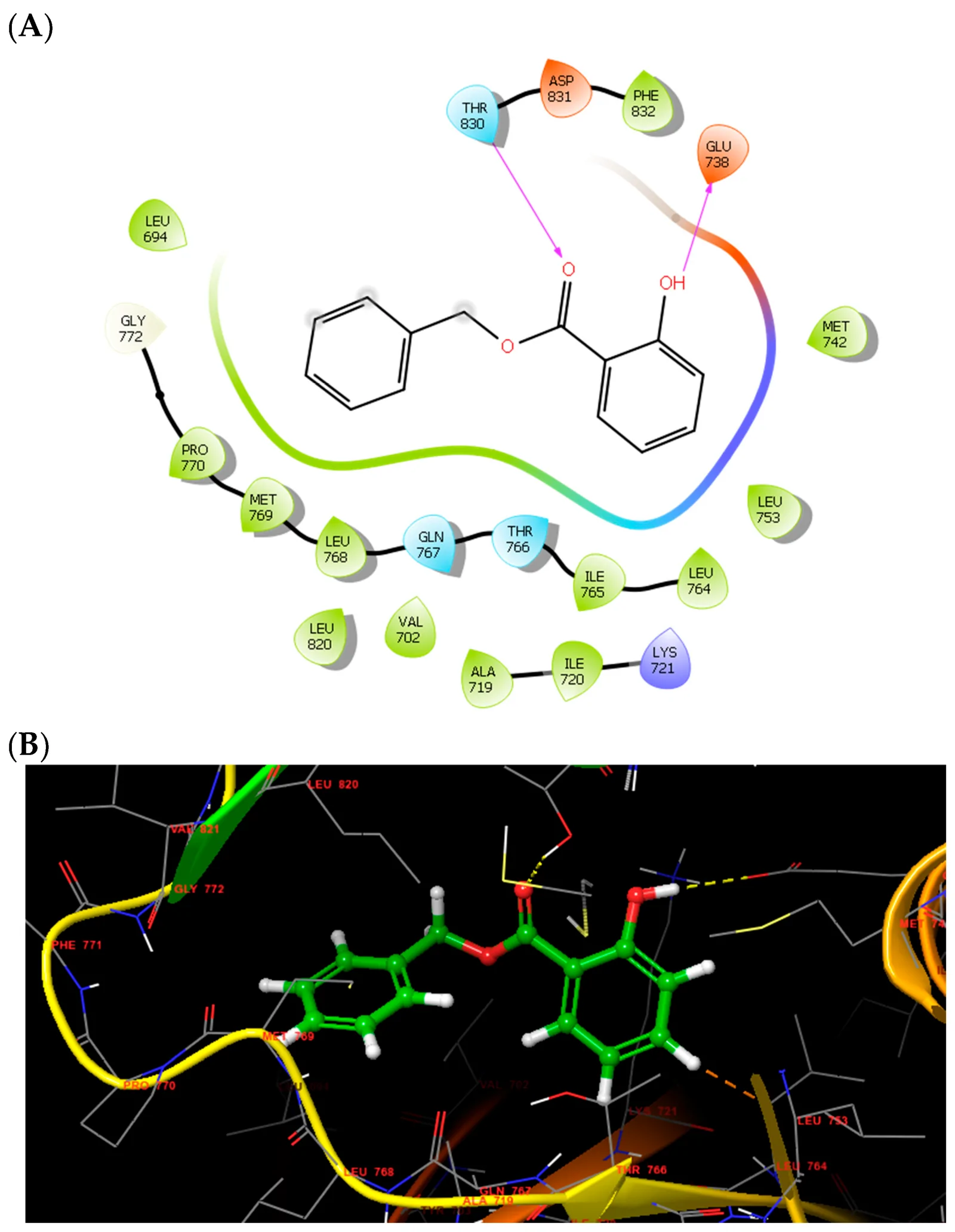
2D (**A**) and 3D (**B**) interaction of benzoic acid, 2-hydroxy-, phenylmethyl ester in the active site of EGFR (PDB ID: 1M17).

**Figure 8:**
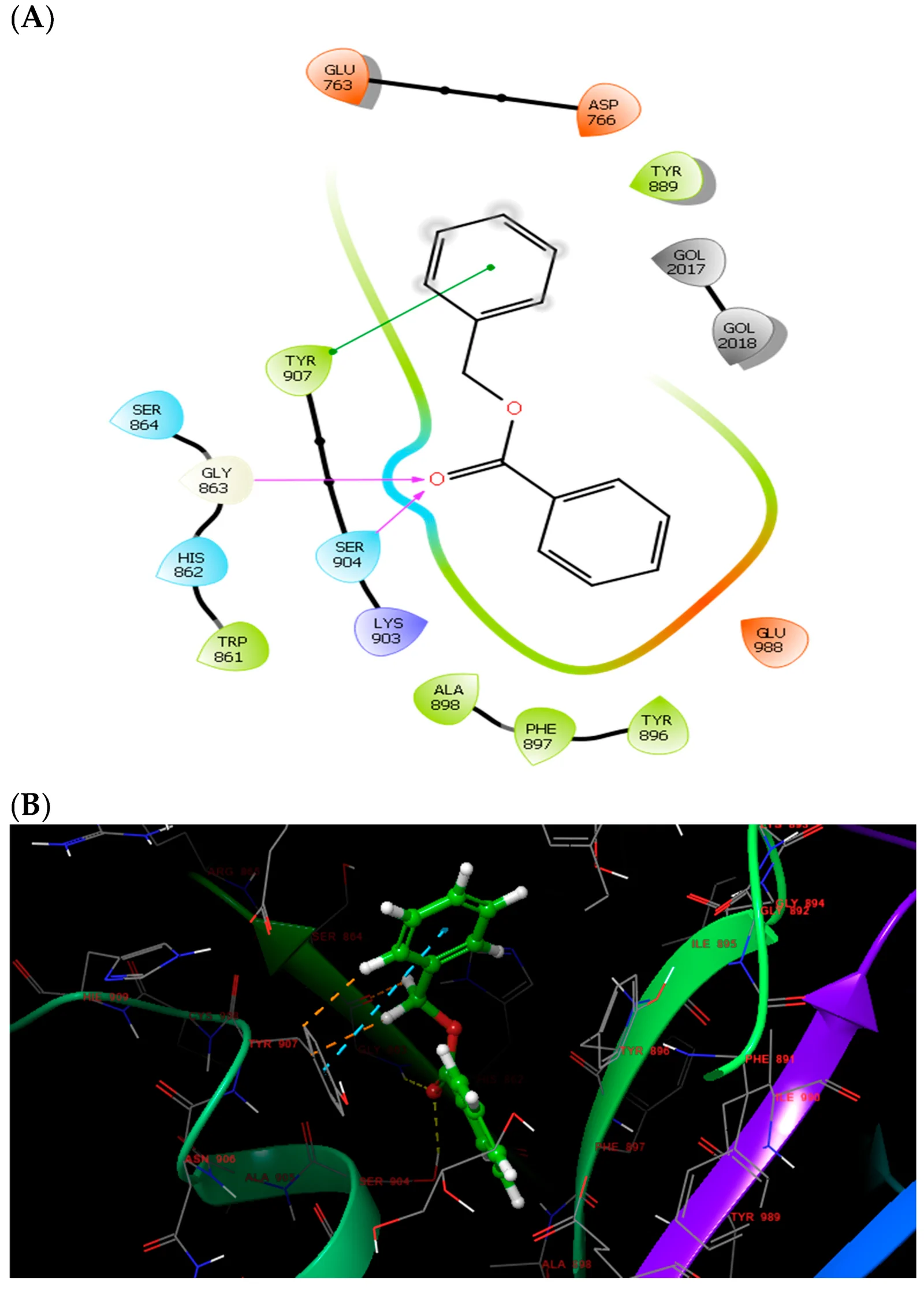
2D (**A**) and 3D (**B**) interactions of benzyl benzoate in the active site of PARP1.

**Table 1:** Main compounds identified in YEO by GC–MS.

Peak	Name	R. Time	I. Time	F. Time	R. Index	Area %	Height %
1	Β-Myrcene	15.947	15.895	16.020	990	0.31	0.35
2	Anisole	17.303	17.230	17.410	1018	1.61	1.58
3	Linalool	19.607	19.530	19.700	1096	4.85	5.52
4	Methyl Benzoate	19.795	19.700	19.880	1090	1.00	1.01
5	Benzyl acetate	21.804	21.735	21.890	1162	1.33	1.47
6	Geraniol	24.229	24.170	24.300	1252	0.46	0.52
7	Geranyl acetate	28.137	28.060	28.230	1381	3.82	4.19
8	Copaene	28.603	28.530	28.680	1376	1.57	1.62
9	Eugenol	28.911	28.850	28.980	1359	0.60	0.65
10	Β-Copaene	30.009	29.940	30.050	1432	1.65	1.68
11	Caryophyllene	30.126	30.050	30.225	1466	15.17	14.97
12	Β-Farnesene	30.455	30.415	30.545	1442	1.58	1.53
13	γ-Muurolene	31.058	31.000	31.120	1479	1.92	2.01
14	α-Humulene	31.221	31.120	31.305	1454	4.83	4.78
15	γ-Cadinene	31.607	31.420	31.680	1513	13.22	12.25
16	α-Cadinene	31.741	31.680	31.815	1538	1.23	1.20
17	Germacrene D	31.926	31.815	31.965	1485	11.97	11.50
18	α-Farnesene	31.990	31.965	32.100	1505	6.45	7.70
19	Ylangene	32.252	32.190	32.390	1420	6.10	4.65
20	Calamenene	33.015	32.895	33.095	1529	1.39	1.45
21	α-Muurolene	33.411	33.355	33.490	1500	0.61	0.66
22	Cubenol	35.976	35.920	36.010	1619	0.35	0.38
23	Junenol	36.053	36.010	36.150	1622	0.60	0.55
24	Tau-Cadinol	36.311	36.240	36.330	1640	0.95	1.13
25	Tau-Muurolol	36.373	36.330	36.490	1642	2.50	1.95
26	α-Cadinol	36.685	36.605	36.780	1654	3.34	3.33
27	trans-Geranylgeraniol	37.688	37.630	37.765	1710	0.61	0.68
28	Benzyl Benzoate	39.519	39.430	39.610	1760	7.84	8.19
29	Farnesol	40.262	40.200	40.335	1743	1.08	1.31
30	Benzyl salicylate	41.881	41.815	41.960	1865	1.10	1.19
	**Total**					**100.00**	**100.00**

**Table 2:** IC50 values and preliminary PBMC-based selectivity indices of YEO and cisplatin in breast-cancer cell lines.

Treatments	IC_50_ Value ± SD (µg/mL) *				Selectivity Index **		
	MCF-7	MDA-MB-231	MDA-MB-436	PBMC	MCF-7	MDA-MB-231	MDA-MB-436
YEO	4.03 ± 0.23 ^a,##^	59.15 ± 3.99 ^b,####^	5.87 ± 0.76 ^a,#^	846.40 ± 22.84 ^c,####^	209.9	14.3	144.2
Cisplatin	7.68 ± 0.88 ^a^	4.68 ± 0.67 ^ab^	3.86 ± 0.58 ^b^	24.28 ± 1.38 ^c^	3.2	5.2	6.3

* Values were obtained from three independent experiments (biological replicates, each performed in technical triplicate) and are expressed as means ± SD (*n* = 3). Within each row, IC_50_ values sharing a superscript letter do not differ significantly (pairwise extra sum-of-squares F-test on log IC_50_, Bonferroni-corrected, *p* ≥ 0.05). ^#^ *p* < 0.05, ^##^ *p* < 0.01, ^####^ *p* < 0.0001: YEO vs. cisplatin for the same cell type (extra sum-of-squares F-test on log IC_50_). ** Preliminary selectivity index (SI) = IC50 in PBMCs/IC50 in the corresponding tumor-cell line. Because PBMCs are biologically distinct from normal mammary epithelial cells, these values should not be interpreted as a definitive therapeutic index or as a direct measure of breast-tissue selectivity.

**Table 3:** Top 5 ligands for each receptor based on composite Gnina score.

Receptor	Ligand	CNNScore	CNNAffinity	Composite Score
ER-α (3ERT)	trans-Geranylgeraniol	0.7831	5.652	4.426
	Farnesyl-decanoate	0.5424	6.695	3.631
	cis-β-Copaene	0.7512	4.624	3.474
	Caryophyllene	0.697	4.801	3.346
	α-Farnesene	0.8438	3.958	3.34
EGFR (1M17)	Farnesyl-decanoate	0.5892	5.962	3.513
	α-Muurolene	0.6269	4.741	2.972
	cis-β-Copaene	0.6366	4.588	2.921
	γ-Cadinene	0.5638	5.077	2.862
	τ-Muurolol	0.5738	4.969	2.851
PARP1 (4ZZZ)	Linalool	0.8629	4.241	3.66
	α-Muurolene	0.6993	5.211	3.644
	cis-β-Farnesene	0.8161	4.179	3.41
	α-Farnesene	0.8275	4.1	3.393
	Benzyl-acetate	0.8589	3.91	3.358

**Table 4:** Docking results of ligands in the different receptors.

	Glide Gscore (kcal/mol)		
Molecule	ER-α (PDB ID: 3ERT)	EGFR (PDB ID: 1M17)	PARP1 (PDB ID: 4ZZZ)
(-)-γ-Cadinene	−5.112	−7.776	−6.864
(1R,4aS,8aR)-4,7-dimethyl-1-propan-2-yl-1,2,4a,5,8,8a-hexahydronaphthalene	−5.209	−7.774	−6.435
(E,E)-3,7,11-Trimethyl-2,6,10-dodecatrienyl decanoate	−4.353	−4.747	−5.426
.α.-Cadinol	−5.821	−8.218	−7.117
.α.-Humulene	−4.972	−7.983	−5.49
.β.-Myrcene	−2.608	−3.922	−2.656
.γ.-Muurolene	−4.845	−7.819	−6.427
.τ.-Cadinol	−5.616	−7.944	−6.164
.τ.-Muurolol	−5.128	−7.77	−6.038
1,2,4a,5,6,8a-Hexahydro-4,7-dimethyl-1-(1-methylethyl)naphthalene	−5.855	−7.951	−6.795
1,6,10-Dodecatrien-3-ol, 3,7,11-trimethyl-, [S-(Z)]-	−2.67	−4.245	−3.017
3-Methoxytoluene	−4.997	−5.853	−6.13
4a(2H)-Naphthalenol, 1,3,4,5,6,8a-hexahydro-4,7-dimethyl-1-(1-methylethyl)-, (1S-(1α,4β,4aβ,8aα))-	−6.218	−7.763	−7.139
α-Farnesene	−1.588	−2.984	−2.07
Benzoic acid, 2-hydroxy-, phenylmethyl ester	−6.573	−7.302	−6.063
Benzyl acetate	−5.516	−6.296	−6.418
Benzyl Benzoate	−6.262	−7.755	−8.058
Bicyclo(5.3.0)decane, 2-methylene-5-(1-methylvinyl)-8-methyl-	−5.505	−7.812	−6.349
Caryophyllene	−5.219	−7.928	−5.269
cis-β-Copaene	−3.683	−7.703	−6.058
cis-β-Farnesene	−0.914	−2.241	−0.822
Copaene	−3.707	−7.817	−5.926
δ-Cadinene	−5.907	−8.278	−5.773
γ-Muurolene	−4.845	−7.819	−6.427
Geraniol	−3.526	−5.403	−5.134
Geranyl acetate	−5.253	−5.242	−5.214
Germacrene D	−4.86	−8.024	−5.97
Junenol	−5.228	−7.625	−5.808
Linalool	−3.718	−4.56	−4.397
Methyl Benzoate	−5.187	−5.979	−7.552
trans-Geranylgeraniol	−4.628	−5.164	−3.054
Ylangene	−4.919	−7.374	−5.785

**Table 5:** ADMET properties and drug-likeness evaluation of the ylang-ylang essential oil constituents.

Compound	Formula	MW (g/mol)	QPlogPo/w	H-Donors	H-Acceptors	Ro5 Violations	Oral Absorption
(-)-γ-Cadinene	C15H24	204.35	5.548	0.0	0.0	1	1
(1R,4aS,8aR)-4,7-dimethyl-1-propan-2-yl-	C15H24	204.35	5.549	0.0	0.0	1	1
(E,E)-3,7,11-Trimethyl-2,6,10-dodecatrienyl decanoate	C25H44O2	376.60	8.244	0.0	2.0	1	1
α-Cadinol	C15H26O	222.37	4.035	1.0	0.75	0	3
α-Humulene	C15H24	204.35	5.190	0.0	0.0	1	1
β-Myrcene	C10H16	136.23	4.601	0.0	0.0	0	3
γ-Muurolene	C15H24	204.35	5.585	0.0	0.0	1	1
τ-Cadinol	C15H26O	222.37	3.963	1.0	0.75	0	3
τ-Muurolol	C15H26O	222.37	4.091	1.0	0.75	0	3
1,2,4a,5,6,8a-Hexahydro-4,7-dimethyl-1-(1-methylethyl)naphthalene	C15H24	204.35	5.410	0.0	0.0	1	1
1,6,10-Dodecatrien-3-ol, 3,7,11-trimethyl-, [S-(Z)]-	C15H26O	222.37	4.934	1.0	0.75	0	3
3-Methoxytoluene	C8H10O	122.16	2.314	0.0	0.75	0	3
4a(2H)-Naphthalenol, 1,3,4,5,6,8a-hexahydro	C15H26O	222.37	3.723	1.0	1.70	0	3
Benzoic acid, 2-hydroxy-, phenylmethyl ester	C14H12O3	228.24	3.384	0.0	1.75	0	3
Benzyl Benzoate	C14H12O2	212.24	3.571	0.0	2.00	0	3
Benzyl acetate	C9H10O2	150.17	2.126	0.0	2.00	0	3
Bicyclo(5.3.0)decane, 2-methylene-5-(1-methylvinyl)-8-methyl-	C15H24	204.35	5.522	0.0	0.0	1	1
Caryophyllene	C15H24	204.35	5.126	0.0	0.0	1	1
Copaene	C15H24	204.35	5.437	0.0	0.0	1	1
Geraniol	C10H18O	154.25	2.612	1.0	1.70	0	3
Geranyl acetate	C12H20O2	196.29	3.496	0.0	2.00	0	3
Germacrene D	C15H24	204.35	5.474	0.0	0.0	1	1
Junenol	C15H26O	222.37	3.649	1.0	1.70	0	3
Linalool	C10H18O	154.25	3.140	1.0	0.75	0	3
Methyl Benzoate	C8H8O2	136.15	2.119	0.0	2.00	0	3
Ylangene	C15H24	204.35	5.415	0.0	0.0	1	1
α-Farnesene	C15H24	204.35	6.992	0.0	0.0	1	1
cis-β-Copaene	C15H24	204.35	5.347	0.0	0.0	1	1
cis-β-Farnesene	C15H24	204.35	6.615	0.0	0.0	1	1
δ-Cadinene	C15H24	204.35	5.356	0.0	0.0	1	1
γ-Muurolene	C15H24	204.35	5.585	0.0	0.0	1	1
trans-Geranylgeraniol	C20H34O	290.50	5.961	1.0	1.70	1	1

## Data Availability

Data will be available upon request from the corresponding author.
